# Views on a brief mindfulness intervention among patients with long-term illness

**DOI:** 10.1186/s40359-016-0163-y

**Published:** 2016-11-15

**Authors:** Ana Howarth, Linda Perkins-Porras, Claire Copland, Michael Ussher

**Affiliations:** 1 Population Health Research Institute, St George’s University of London, London, UK; 2 Institute of Medical and Biomedical Education, St George’s University of London, London, UK; 3 Chronic Pain Service, St George’s University Hospitals NHS Foundation Trust, London, UK

**Keywords:** Chronic illness, Brief intervention, Mindfulness, Qualitative analysis

## Abstract

**Background:**

Chronic illness is the leading cause of death in the UK and worldwide. Psychological therapies to support self-management have been shown to play an important role in helping those with chronic illness cope; more recently, the therapeutic benefits of mindfulness approaches have become evident for managing depression and other distressing emotions. Brief guided mindfulness interventions, are more convenient than intensive traditional programmes requiring regular attendance but have been less explored. This study assessed views on a brief (i.e., 10 min) mindfulness intervention for those with specific long-term illnesses.

**Methods:**

Semi-structured interviews and focus groups were conducted with chronic illness patient groups (i.e., chronic obstructive pulmonary disease, chronic pain and cardiovascular disease), designed to capture the acceptability and feasibility of the intervention. The interviews were conducted after use of a mindfulness based audio in clinic and, one week later, after use in the patient’s own environment. Interviews were recorded, transcribed and analysed using thematic analysis.

**Results:**

In total, a combination of 18 interviews and focus groups were conducted among 14 patients. Recruitment was most successful with chronic pain patients. All patients reported benefits such as feelings of relaxation and improved coping with symptoms. While the wording and content of the audio were generally well received, it was suggested that the length could be increased, as it felt rushed, and that more guidance about the purpose of mindfulness, and when to use it, was needed.

**Conclusions:**

A brief mindfulness intervention was well accepted among patients with long-term illness. The intervention may benefit by being lengthened and by offering further guidance on its use.

**Electronic supplementary material:**

The online version of this article (doi:10.1186/s40359-016-0163-y) contains supplementary material, which is available to authorized users.

## Background

Globally, chronic illness is the leading cause of death [1]. Moreover, based on WHO estimates, the burden of chronic illness is expected to increase 57 % by 2020 [1]. In the UK, the National Health Service (NHS) budget is devoted to chronic disease care, with approximately £7 of every £10 spent on patient care going towards chronic illness. Cardiovascular disease (CVD), chronic pain and chronic obstructive pulmonary disease (COPD) are some of the most common chronic illnesses. In the UK, CVD accounts for over 150,000 deaths a year and affects more than five million people, with annual costs exceeding £30bn [2]. Chronic pain has a negative impact on quality of life [3], resulting in high levels of disability [4], with high comorbidity rates of depression and anxiety [5]. Within the COPD population, anxiety has been linked to greater disability, and increased frequency of hospital admissions for acute exacerbations and dyspnea [6, 7].

Mindfulness-based interventions have been shown to have benefits for the management of many common psycho-social issues associated with chronic illnesses, including anxiety, depression, distress and quality of life [8]. Mindfulness can be defined as “paying attention in a particular way: on purpose, in the present moment and non-judgementally.” [9]. Conventional medicine has recognised the benefits of mindfulness–based interventions; for example, recent NHS guidelines recommend mindfulness meditation for depression [10].

While this research is promising, a major barrier with the implementation of current mindfulness interventions is the investment of time they require, which is typically eight weekly group-based sessions, on top of daily home practice. Limited physical and mental resources, make brief interventions likely to be acceptable to chronic illness patients. The self-management model of care [11] is now integral to the NHS and has led to an emphasis on the development of brief interventions and self guided programmes.

One type of brief intervention that fits this profile is a short mindfulness-based body scan. These scans are a key component of mindfulness meditation; they involve being directed to focus attention on the present moment through observing the breath, and bodily sensations, while becoming aware of, and accepting without judgment, any thoughts and feelings which arise. Traditional mindfulness based interventions routinely include a body scan meditation, lasting anything from 5 to 45 min [12].

In healthy populations, during experimental pain studies, brief mindfulness interventions, have been successful in reducing some aspects of the pain experience, such as distress and sensitivity [13, 14]. A brief mindfulness intervention with heart disease patients having recently undergone surgical treatment found increases in social quality of life and decreased perceived anxiety and stress [15]. In a chronic pain population, a 10 min body scan reduced reports of distress in a clinical setting [16]. Together, these findings provide preliminary support for further investigating a brief mindfulness intervention as a self-management tool within chronic illness populations.

The current study assessed the views of patients with chronic illness regarding the acceptability of a brief mindfulness-based intervention as a self-management tool, so as to refine the intervention, define the suitability of the intervention for patients with different chronic illnesses (including ease of recruitment), and to design a process for intervention delivery and assessment.

## Methods

Reporting of the study was guided by the Consolidated criteria for reporting qualitative studies (COREQ): 32-item checklist [17]. The checklist itself is attached in Additional file 1.

### Participants

Three chronic illness populations were recruited: patients with COPD, CVD or chronic pain, attending outpatient clinics at St George’s University Hospitals NHS Foundation Trust, were screened by the relevant consultant for eligibility. To be eligible patients needed to be well enough to participate according to the referring consultant, able to speak and read English, able to hear the audio recording, and were at least 18 years of age. The aim was to recruit around six patients in each illness group.

### Interview methodology

Semi-structured interviews and focus groups were conducted using a topic guide (see Additional file 2), which focused on how using the audio recording made participants feel, how acceptable they found the content and presentation, how useful they thought the recording might be to manage chronic illness symptoms, and any suggestions they had for improving the intervention.

The audio itself was a 10 min body scan based on a transcript from Breathworks an established mindfulness organisation specialising in supporting those with chronic pain. Loaded onto an MP3 that patients could take home to use in their own environment, the body scan consisted of directions for the listener to ‘scan’ their body with their attention systematically, starting with the toes and finishing with the crown of the head. Throughout this process, the listener was also encouraged to be aware of their breathing and to accept all thoughts and feelings, whether positive or negative, without trying to alter them in any way. Administered face-to-face for the initial use by a researcher in a clinical setting (i.e., private interview room), patients were requested to use of the audio at least three further times during the following week before returning for a follow up focus group interview.

Questioning opened with the line “To begin, I would like to ask you to describe your previous experience with yoga, tai-chi or any type of meditation.” Information gathered in early interviews informed subsequent interventions. All interviews were face-to-face and conducted in a private interview room at St George’s, University of London by the first author who had previous qualitative project experience. Prior to starting the interview, the interviewer explained the reasons for conducting the research as well as their personal interest in the study. An initial interview was conducted after the patient had listened to the audio recording of a 10 min guided mindfulness body scan, in clinic with the researcher. The intention was to conduct a focus group, one for each chronic illness population, approximately one week later after the patient had used the recording, in their own home, at least two more times.

To assess patient word preference for labeling the intervention, focus groups were concluded with a short discussion of potential names based on a list of fourteen options suggested by experts. These names were: stress reduction, attention training, relaxation training, coping training, chronic illness coping, focused relaxation, attention management training, cognitive focus training, mindfulness meditation, relaxation audio-guide, body and breathing scan, body scan, breathing scan and breathing exercises. Again, as the interviewing progressed, feedback from patients informed subsequent interviews.

Prior to the interviews, written consent was obtained from patients. Interviews were recorded with digital voice recorders, which were transcribed verbatim. Ethical approval was given by the Office for Research Ethics Committees Northern Ireland (ORECNI), REF 14/LO/1912.

### Analysis

Analysis of the transcripts was conducted using thematic analysis, which is considered appropriate for analysis of participant views within clinical and health care settings (Marks & Yardley, 2004). All transcripts were read by the main investigator (AH) as well as additional researchers (MU, JS and ML), who were not involved with the interviewing and therefore offered potential insurance against bias.

The categories for initial classification were derived from the topic guides for the interviews. Using the qualitative data analysis software, QSR International’s NVivo 10, the researchers reviewed the transcripts individually and allocated data to corresponding relevant key topics (i.e. nodes in NVivo). These key topics with allocated data were then individually analysed and coded, which provided a basis for the development of emerging themes.

After researchers had independently produced a set of themes for each key topic, the researchers met, and so as to establish inter-rater reliability, discussed, agreed upon and refined the themes. The refined themes were then compared across the key topics and if overlapping, developed into broader themes that more comprehensively presented the data in a way that addressed the original research aims.

## Results

### Recruitment

Four consultants, specialising in either chronic pain, CVD or COPD, screened patients for referral between May 2014 and Jul 2014. Patients were either referred in clinic when the researcher was present or, if the researcher was not present, details of patients’ who agreed to be contacted, were passed to the researcher. Thirty-five patients in total were eligible and were referred to the researcher. Of these, 14 patients (aged 21–78 years) agreed to participate of which six had chronic pain, five COPD and three CVD. Between June and August 2014, 14 participants were individually interviewed, 13 of these were followed up in focus groups, and one individual was followed up by interview (due to a personal matter).

#### Cardiovascular disease population

Among 50 patients attending clinics, one consultant referred 11 patients and three of these participated (two females, aged 54 and 67; one male, aged 52).

#### COPD population

Two consultants referred COPD patients. Of 30 patients attending his clinics, the first consultant referred five patients and one patient agreed to participate. The second consultant facilitated an introduction to a rehabilitation clinic for COPD patients and, out of 12 patients attending, four patients were referred and four were recruited. The latter patients were already visiting the clinic weekly and therefore research sessions were combined with these visits. The final group consisted of two females (aged 31 and 63) and three males (aged 55, 65 and 78).

#### Chronic pain population

Among 20 patients attending clinics, one consultant referred 12 patients and six participated (five females aged 21, 55, 57, 58 and 69 and one male aged 47).

#### Sample characteristics across populations

Across the three patient groups, there were some similarities in both the referral process and the engagement of the patients. It was noted that consultants, often reported tending to deviate from the eligibility criteria by referring patients who were in need of psychological support or had more obvious psychological co-morbidity. Patients who were positive when approached about participating but ended up not consenting, often reported the main reason was not being well enough or not having the resources to attend two research appointments. Across the final population that enrolled, almost two thirds had no previous experience with activities similar to the intervention, such as yoga, tai chi or meditation.

### Thematic analysis of interview responses

The initial classification of patient views was based on the key topics outlined in the topic guides, i.e.: perceived benefits of the audio, acceptability of the audio and improvements to the audio. As similar themes emerged from both interview and focus groups, all data was combined and five main themes emerged: perceived benefits of the audio, negative experience of the audio, content of the audio, perceived barriers and improvements suggested. As presented in Table 1, these main themes resulted in a total of twelve themes and sub-themes altogether. Assessment of preferred intervention names was analysed separately.

**Table 1:** Key topics and theme development

Key Topics as defined in Topic Guide	Refined Themes and sub-themes
Perceived benefits of the audio	1. Perceived benefits of the audio • *Relaxation* • *Mood improvement* • *Increased coping abilities* • *Reduction in use of medication* • *Increased motivation towards meditation*
Acceptability of the audio	2. Negative experiences of the audio 3. Content of the audio • *Wording of the audio* • *Timing and Pace* • *Voice of the narrator* 4. Perceived barriers
Improvements to the audio	5. Improvements suggested • *Timing and Pace improvements* • *Presentation*

### 1. Perceived benefits of the audio

Perceived benefits were one of the most notable themes due to the large positive response. Five sub-themes emerged under this main theme:

#### Relaxation

Relaxation was the most frequently mentioned benefit of the audio, with such terms as ‘slowed down’ or ‘calmed’ often used:

*C12* 1 *: But I feel very relaxed now, you know? Well, I would say, from just listening to that today, that did relax me. I feel slower!*

Sometimes the relaxed feeling was a specific physical sensation:

*B08: I noticed that towards the end of it, I was actually loosening up, where normally I’m not. I could actually feel relaxed and even helping my arthritis joints and stuff like that, because it was going through the joints. So I found that very helpful…*

#### Mood improvement

Positive mood was often reported immediately after listening to the audio:

*A06: I felt a slight euphoria, with that sort of, erm, having taken time out for yourself kind of feeling which you so rarely do, but when you do, you think, you know, it’s almost like a self-rewarding thing, which is great.*

#### Increased coping abilities

The increased ability to cope with a variety of stressors was commonly reported as a perceived benefit, often felt immediately after using the audio. Sometimes this increased ability was during an episode of acute symptoms:

*A03: Yeah, and my breathing calmed down and it was more, as the pain was … because of my breathing calming down, it eased it off.*

*B08: See? So it’s like you notice … my chest is tightening but you’re not getting into a panic.*

Sometimes patients reported that their ability to cope with stressful everyday life situations in general was aided:

*A04: Yes, to switch off from that tension that you have in your head*

*B10: I always have trouble sleeping anyway, because I live down a really noisy road that’s got lovely buses, you know?*

*I: How did the audio go then with the buses?*

*B10: It didn’t quite bother me so much, especially in the evening.*

Overall, patients felt confident recommending the audio to increase coping ability:

*B12: I think it would be good for anybody with stress, definitely stress, and pain management.*

*B02: I’d had a really hard day at work. So, got home and listened to the tape, and again it made me feel a good deal more relaxed and a bit more positive about the world.*

#### Reduction in use of medication

An unexpected finding that should of course be interpreted cautiously, is the spontaneous reporting by some patients of being able to reduce their use of medication. This behavior was not suggested at any point by the researcher, but as the dependency and side effects of long term medication use is often problematic, the findings are reported here:

*A03: Well, when I did it, I actually skipped that dose of medication, so I think if I do that for that dosage, then I can cut back on however many tablets I take a day, which is quite a few.*

*C13: I’ve got asthma and I found that helped, helped me breathe a bit better, a bit clearer, you know? I weren’t taking my inhaler so much.*

#### Increased motivation towards meditation

One final notable benefit, reported by several participants, was an increased motivation towards practicing meditation:

*A01: And it’s good because it might start me off again with actually doing it [meditation practice] on a regular basis …*

*C12: Yeah, because the more you do it, the better the feeling I think, anyway.*

### 2. Negative experience of the audio

Overall, patients had an overwhelming positive response to listening to the audio but some negative experiences were also reported. These were mainly in relation to discomfort in their areas of vulnerability, either when they were directed to give awareness to an area of pain or just spontaneously.

*A03: When it mentioned the arms, because my pain is in my right arm, I actually started getting uncomfortable; I was distracted up until that point, and then I was like, oh, god, now I’m uncomfortable. And then I started to fidget a little bit, and that happened all three times.*

*C12: I think because where it says put your mind onto your chest, it made my chest ache, heavy.*

### 3. Content of the audio

When questioned about aspects of the content, such as the wording, instructions or narrative, patient assessments were generally positive. They appeared to be able to follow what the audio guide was asking them to do. There were three sub-themes under this theme of content: wording of the audio, timing and pace of the audio and voice of the narrator.

#### Wording of the audio

The audio begins with an explanation that the audio is for the patient to relax and take some time for themselves. This was meant to pave the way for the mindfulness content which may have been a new experience; overall the introduction appeared to be sufficient in doing this.

*Did you feel reassured when you started out, that it was going to make sense? Did it kind of flow?*

*B07: Yeah. I can’t think of anything that didn’t make sense. It made sense.*

The main body of the audio included some mindfulness wording and instructions on how to conduct the body scan, which appeared to be well received in most cases:

*A06: Work methodologically through the body, non-judgemental, not putting words into your psyche, as it were. So, yeah, it was good, I enjoyed it.*

Some of the mindfulness content focused on a key component of mindfulness meditation, which is simply bringing attention to the breath. While this was a new concept for some patients, they offered positive feedback.

*A03: When I started getting uncomfortable, I thought, no, I’ll do what the tape says! And bring myself back to the voice.*

Overall, the presentation of the narrative was reported to be quite acceptable, if not enjoyable:

*A03: It was like not having to actually do anything, just listen is the easiest thing to do, so I liked that.*

#### Timing and Pace

Overall, patients responded positively when asked about the timing and pace of the audio. Comments relating to improvements are reported within the ‘improvements suggested’ theme.

*A05: No, I think it was just about right. Perhaps it could be a tiny bit slower, but I thought it was all right.*

*B01: No, I think the pacing is ideal. Because if you do anything too fast, it doesn’t work. You can’t hurry it, full stop; where the mind is concerned, you’ve got to take it easy.*

#### Voice of the narrator

Overall, the voice appeared to be appropriately pitched. In some instances, the voice was reported as soothing and potentially facilitated the use of the audio:

*A06: Yeah. It was a very nice voice actually, I enjoyed it… it was nice, again a sort of round, relaxed and sort of caring voice, which is what you’re after.*

*C12: I think the voice was very calming and nicely spoken, and not rushed.*

### 4. Perceived barriers

Despite being asked what or why things might get in the way of using the audio, patients mostly responded that they could not see anything getting in the way, especially because the time needed was minimal:

*C12: And even time, because ten minutes is not much out of anyone’s time, is it?*

The one barrier that was reported by one patient was timing, or really the planning of using the audio:

*A06: You may have the time but it’s about prioritising it, so it’s how can you make it come up in your daily activities*

### 5. Improvements suggested

When asked what could be improved upon, two main sub-themes emerged: timing and pacing and presentation of the audio.

#### Timing and pacing improvements

Patients suggested that a little more time might improve the audio.

*A01: I think it’s too fast. (lots of general agreement)… I think she needs to slow down and maybe that might make it seem a bit more calming. (lots of general agreement).*

*A02: And I think it’s good that it’s short, but as far as helping with pain, as I said earlier, I think you need to allow a little more time.*

It soon became apparent that 15 min would be a more acceptable length, so this option was then introduced in subsequent interviews. Patient views on extending the length of the audio to 15 min were very positive:

*A02: I don’t think 15 minutes is too much to ask. By the time I was up one arm, she was onto the next thing and I hadn’t left that arm, and she was onto the leg!*

*B08: I think 15 minutes is, like, just right, because otherwise it’s like, erm, it’s crowding you.*

#### Presentation

Although the researcher introduced the audio to the patient during the first session, some aspects of the mindfulness wording or narrative were found to be slightly confusing.

*A05: The bit about sort of breathing into the pain, or breathing into the area that hurts, I didn’t understand that.*

Based on this feedback, the researcher subsequently enquired about the usefulness of having someone present when listening to the audio for the first time and the overwhelming response was that this was vital:

*C12: Well, I’d say our meeting, having that first meeting was good to sort of introduce us to it, because I wouldn’t have a clue on anything like that.*

Following this, the researcher asked if it might be helpful if some further explanation of mindfulness accompanied the audio, and this was enthusiastically endorsed.

*A03: I think if a pamphlet came with the tape, just to identify maybe certain terminologies for the average person.*

It was also noted that it would be useful to mention that the audio may have accumulative effect, with it being more effective following more regular use:

*A02: You know, that the first time, or the first couple of times, it’s not going to, and the best way is the accumulative effect of it and doing it regularly.*

Encouraging usage at any time and in any position was a further suggestion:

*A03: If it was phrased as ‘do it when you need it!’. That would be better than to say ‘Do it three times a day!’, because when you need it, it will probably be around three times a day anyway*

Finally, variety appeared to be another aspect of presentation that could be improved upon. The use of different types of audios (e.g., audios focused on movement or just breathing) was recommended:

*A06: If you’re doing something a long time, you might want a bit of variety, you know.*

### Intervention name

Patient views on intervention names were assessed with a word preference questionnaire. Patients were given a brief list of potential names for the intervention and then asked to discuss their preferences.

Names related to relaxation or referencing stress management were most popular. Training, cognition and attention names were the least popular:

*C12: Yeah, I think the best one out of mine would be relaxation audio guide.*

*C13: When you say ‘training’ it looks like you’ve got to work at it.*

As an alternative, the word ‘coping’ appeared to be acceptable, not in the original way it was used in the list (i.e., chronic illness coping), but instead as the primary word in the title. When this was mentioned in two out of the three focus groups, there was strong concordance:

*A02: Why not use the word coping?*

*A03: Just say, “coping with your symptoms.”*

*B08: You’ve got one here, right, it’s got: Chronic illness coping, but if you changed that around slightly and that’s: coping with chronic illness, would actually be …*

*I: A coping with illness audio?*

*B10: Yeah, because that just doesn’t sound right to me, you know, but putting it the other way around sounds a bit better. Because everyone wants to cope with their illnesses, don’t they?*

## Discussion

### Feasibility of recruitment

Many patients were willing to participate but two factors limited participation. The first was travel, which was a major barrier as patients were often not physically well enough to attend two clinic sessions. In particular, the CVD and COPD population patients had high comorbidity rates, which substantially decreased ability to participate. This could be remedied in future by limiting the intervention to one session in clinic, by offering home visits or by coinciding the research visits with routine appointments, as was done with some of the COPD patients. The second factor influencing participation was dependence on referrals from consultants. Recruitment solely through consultants was not ideal as most patients were recruited when they had the opportunity to meet the researcher after consultations. Meeting the researcher in person appeared to be more reassuring and motivating.

Finally, following recruitment, it became clear that there had been lack of transparency regarding consultant’s inclusion and exclusion criteria for referral. In future, consultants may need more explanation to justify the agreed criteria and to appreciate the importance of discussing with the researcher if they feel the need to deviate from the criteria.

### Population suitability

The chronic pain population was the easiest group to recruit due to consultant support and population characteristics such as lower age, ease of mobility and motivation to try interventions to facilitate pain management. Going forward, the results show that this population are most likely to be recruited in sufficient numbers and would be most suited to make an intervention study feasible. Another avenue of recruitment to be considered for patients with chronic pain is physiotherapy clinics.

COPD patients were difficult to recruit due to the volatile nature of their illness (i.e., still in the process of being stabilized) and their older age making travel more challenging. Contrastingly, COPD patients who were in a programme specifically to manage their condition were generally more physically able and motivated to participate, even though the average patient was elderly and had high comorbidity rates.

CVD patients who were referred from clinic were often very positive about volunteering. Unfortunately, due to high comorbidity rates, they were unlikely to be able or willing to attend two sessions and this suggests that they may be difficult to recruit for intervention studies.

#### Acceptability

The qualitative analysis showed that many aspects of the audio intervention were considered very acceptable in both content and presentation. However, several of the suggested improvements to the audio can be used to refine it. The perceived benefit most frequently reported by patients was a feeling of relaxation. Relaxation was described in a variety of ways, including the experience of slowing down or becoming drowsy. Although relaxation is not the main goal of mindfulness, it is often mentioned as a beneficial side effect. Relaxation in relation to the management of illness symptoms is of obvious importance as it works in opposition to anxiety and distress [18], which are two of the key burdens of chronic illness, reducing quality of life. Previous research using a comparable brief mindfulness intervention found an immediate effect in clinic of a significant decrease in pain-related distress [16].

Similarly, patients reported mood enhancement and increased coping abilities during and after using the audio during stressful times. For some, this positive change in mood allowed them to take up activity they normally would not have the energy for. For others, the ability to cope better while experiencing acute symptoms was the result. Further benefits with regards to medication reduction were promising as three out the fourteen patients reported successfully reducing or forgoing their medication on certain occasions when using the audio. This was an unexpected benefit and not one that has been recorded in previous research with this type of intervention.

Conversely, while mindfulness encourages bringing awareness to the present moment, some patients reported that bringing awareness to their bodies was uncomfortable at times, as they were being asked to bring attention to an area that may have pain or distressing symptoms. Fortunately, the same patients reported that this was not an unacceptable experience, they did not feel overcome and, while listening to the audio, they were able to move on without the panic and distress that often accompanies these symptoms. In future, it may be useful to add instructions about how some feelings or experiences may be uncomfortable.

These results, along with the generally positive patient response to the content of the audio, suggest that the wording, guidance and narrative of the audio were acceptable. The voice was considered unobtrusive at worst and most patients said that it was an easy audio to listen to with instructions that were easy to follow.

### Practical implications

When questioned about specific changes that could be implemented to make the audio more likely to be used, the main feedback was that it was too rushed and could be longer. All but one in the pain group (i.e., the largest group) said that a slightly longer audio of 15 min would be preferred. Previous studies using brief mindfulness interventions used audios ranging from 5 to 20 min [19–22], with varying effects. Experimental studies are needed to compare the relative effects of different durations of mindfulness body scans.

It was agreed that it was important that the researcher had introduced the audio. All participants reported that this introduction should be with someone knowledgeable about the intervention, as it was reassuring and a source of motivation. Patients also reported that having a leaflet or information sheet about mindfulness to take home would have been helpful when using the audio on their own. Other studies using brief mindfulness interventions have not provided any information alongside the intervention but each use was under the guidance of either an instructor or researcher [13, 23].

One area that inspired debate was the intervention name. The main feedback was that mindfulness as a name for the intervention made sense once it had been explained but that, when unfamiliar, labels such as relaxation and stress reduction may be most apt possibly because mindfulness as a practice is still new for many or perhaps because the relaxation effects of the intervention were the most valuable aspect, in the first instance. The names that were most popular overall included anything with coping in the title, followed by whatever the relevant symptom may be (i.e., ‘coping with chronic illness”). Investigation of the preferred name or label of this type of intervention has not been reported previously and research has typically referred to this type of intervention as “mindfulness training”, “body scan”, “attention training” and “mindfulness training” [16, 21, 24].

Finally, few perceived barriers were reported. Most patients reported that they could not offer a reason why they might not be able or willing to use the audio for the allocated time. In future, interventions should include a longer recommended time period for use and measures not just after one week but at a later date as well.

### Strengths and limitations

A strength of this study is the provision of detailed data in relation to patients’ views on the acceptability of a brief mindfulness intervention to facilitate coping with chronic illnesses. However, due to patient health complications, the representation of each illness group was not balanced.

High patient numbers may have increased the quantity of data but as the interviews and focus groups were longer in length than initially expected (i.e., some interviews lasted up to 45 min and some focus groups lasted up to 1.5 h), it was felt that the depth of the input per patient allowed for an increase in quality and that data saturation was reached. All interviews and focus groups were conducted by one researcher (i.e. the first author), potentially allowing for bias, but a standardised topic guide was adhered to. It is acknowledged that this qualitative evaluation focusses on the immediate benefits of the intervention and to examine sustained changes to patients everyday lives follow-ups across several months would be required.

## Conclusions

This qualitative study found that recruitment tended to be most successful within a chronic pain population and was most feasible with the support of consultants, alongside face-to-face contact between patient and researcher. The audio based mindfulness intervention was considered to be highly acceptable to patients and the main benefits reported were the immediate, post-intervention, experiences of relaxation and increased mood and enhanced coping with illness symptoms. Although no substantial barriers were reported, the key improvements suggested were to extend the audio to 15 min in length and to include some written explanations of basic mindfulness concepts, as well as guidance about when to use the audio. These qualitative results provide a firm basis for further development and refinement of the intervention.

## Additional files

Additional file 1: COREQ Checklist. (DOCX 20 kb)
Additional file 2: Topic Guided for interviews and focus groups. (DOCX 76 kb)

## Abbreviations

BPI: Brief pain inventory
JREO: Joint Research & Enterprise Office
MBSR: Mindfulness based stress reduction
MP3: MPEG-1 Audio Layer 3
NHS: National Health Service
ORECNI: Office for Research Ethics Committees Northern Ireland

## Footnotes

1 Grouping is according to patient population. Group “A” are chronic pain patients, “B” are COPD patients and “C” are CVD patients and number following is patient ID in that group.
