# Supplementary material for: Views on a brief mindfulness intervention among patients with long-term illness
Source: BMC Psychol. 2016 Nov 15;4:56. doi: 10.1186/s40359-016-0163-y (PMC5109661; doi:10.1186/s40359-016-0163-y)
Supplement: Additional file 2: — Topic Guided for interviews and focus groups. (DOCX 76 kb) [file 40359_2016_163_MOESM2_ESM.docx]

**Topic Guide for Individual Interview**

Background

1. To begin, I would like to ask you to describe your previous experience with yoga,

tai-chi or any type of meditation.

Acceptability

1. What did you think of the audio (e.g. length of the audio, voice and style of the narrator)?
2. If there was a way to improve the audio whether it be content or the way it was delivered, what would you suggest?
3. Did you feel any benefit or difference after listening to the audio?
4. How might this audio be helpful to people living with chronic illnesses?

Feasibility

1. How easy do you think this audio recording might be to you or others with your illness in helping to manage illness symptoms and any feelings of distress?
2. What do you think about the how the audio asked you to focus your attention and how it suggested you try accepting your thoughts and feelings?
3. Is there anything that you can think of that would increase or decrease your motivation to use this type of audio that we haven’t discussed already?
4. How do you think the audio could be applied to your everyday life and what barriers do you see to this audio being useful?
5. What do you think is the best way to describe a mindfulness audio so as to encourage someone to try it out?

**Topic Guide for Focus Group**

Background

1. To begin, I would like to ask you to share with the group any your previous experience with yoga, tai-chi or any type of meditation that you might think is relevant.

Acceptability

1. What did you think of the audio recording (e.g. length of the audio, voice and style of the narrator)?
2. If there were some ways to improve the audio whether it be content or the way it was delivered, what would you suggest?
3. Did you feel any benefit or difference after listening to the audio?
4. How might this audio be helpful to people living with chronic illnesses?

Feasibility

1. How easy do you think this audio recording might be to you or others with your illness in helping to manage illness symptoms and any feelings of distress?
2. What do you think about the how the audio asked you to focus your attention and how it suggested you try accepting your thoughts and feelings?
3. Is there anything that you can think of that would increase or decrease your motivation to use this type of audio that we haven’t discussed already?
4. How do you think the audio could be applied to your everyday life and what barriers do you see to this audio being useful?
5. What do you think is the best way to describe a mindfulness audio so as to encourage someone to try it out?
